# The Role of CENPK Splice Variant in Abiraterone Response in Metastatic Castration-Resistant Prostate Cancer

**DOI:** 10.3390/cells13191622

**Published:** 2024-09-28

**Authors:** Minhong Huang, Sisi Qin, Huanyao Gao, Wootae Kim, Fang Xie, Ping Yin, August John, Richard M. Weinshilboum, Liewei Wang

**Affiliations:** 1 Department of Molecular Pharmacology and Experimental Therapeutics, Mayo Clinic, Rochester, MN 55905, USA; 2 Department of Pathology, University of Chicago, Chicago, IL 60637, USA; 3 Department of Integrated Biomedical Science, Soonchunhyang Institute of Medi-Bio Science (SIMS), Soonchunhyang University, Cheonan 31151, Chungcheongnam-do, Republic of Korea; 4 Division of Medical Oncology and Cancer Center, Beth Israel Deaconess Medical Center, Boston, MA 02215, USA

**Keywords:** metastatic castration-resistant prostate cancer, splice variant, drug resistance, patient-derived xenograft, 3D organoid

## Abstract

Most patients with metastatic prostate cancer eventually develop resistance to primary androgen deprivation therapy. To identify predictive biomarker for Abiraterone acetate/prednisone resistance, we screened alternative splice variants between responders and non-responders from the PROMOTE clinical study and pinned down the most significant variant, CENPK–delta8. Through preclinical patient-derived mouse xenograft (PDX) and 3D organoids obtained from responders and non-responders, as well as in vitro models, aberrant CENPK–delta8 expression was determined to link to drug resistance via enhanced migration and proliferation. The FLNA and FLOT1 were observed to specifically bind to CENK–delta8 rather than wild-type CENPK, underscoring the role of CENPK–delta8 in cytoskeleton organization and cell migration. Our study, leveraging data from the PROMOTE study, TCGA, and TCGA SpliceReq databases, highlights the important function of alternative splice variants in drug response and their potential to be prognostic biomarkers for improving individual therapeutic outcomes in precision medicine.

## 1. Introduction

Prostate cancer is the second most frequent malignancy among men worldwide with over 191,000 new cases diagnosed per year and is estimated to have caused 33,000 deaths in the United States in 2023 [1,2]. Androgen deprivation therapy is the primary systemic treatment for prostate cancer [3], but most patients eventually become resistant to the therapy and develop castration-resistant disease, leading to high mortality. Hence, castration-resistant prostate cancer (CRPC) is a major challenge in clinics to achieve effective treatment outcomes [4]. The development of new generation of androgen receptor (AR) signaling inhibitors, such as the CYP17 inhibitor, Abiraterone, or the AR blocker, enzalutamide, has significantly increased the survival of patients with CRPC [5,6,7]. However, approximately 20% of metastatic castration-resistant prostate cancer (mCRPC) patients still develop either primary or acquired resistance [8,9]. Thus, there has been a considerable effort in attempts to identify response biomarkers and molecular mechanisms underlying the resistance.

In our previous study, we investigated the PROMOTE clinical trial, aimed at identifying resistance signatures to Abiraterone acetate/prednisone (AA/P) in patients with metastatic castration-resistant prostate cancer (mCRPC) at the Mayo Clinic. We conducted exome and RNA sequencing on biopsy samples collected at baseline and 12 weeks after initiating AA/P treatment. We analyzed genomic alterations and differentially expressed genes to compare responders and non-responders, as defined by a 12-week composite score. Our analysis revealed that the genes associated with Wnt signaling and cell-cycle pathways were upregulated in resistant patients [10]. Additionally, RNA sequencing data identified mRNA splicing events in baseline tumors showing significantly associated with AA/P response. Notable splice variants included upregulated KIAA1324, CENPK, and PKN1 variants, as well as downregulated EMD and TSC2 variants in resistant patients. Specifically, the KIAA1324 splice variant, encoding the estrogen-induced gene 121 protein (EIG121) with a 126-nucleotide deletion in Exon 16, was found in 70% of non-responders versus 26.7% of responders (*p* = 3.0 × 10⁻^4^). The CENPK splice variant, with Exon 8 skipping, was observed in 63.3% of non-responders and 28.9% of responders (*p* = 4.3 × 10⁻^3^). The PKN1 splice variant, featuring Exon 8 and 9 skipping, appeared in 76.7% of non-responders compared to 44.4% of responders. Conversely, the TSC2 and EMD splice variants were significantly under-expressed in non-responders. These findings prompted the current study to investigate these five splice variants further.

In this study, we further investigated these five splice variant biomarkers to pin down the significant ones that attributed to AA/P resistance. Experimentally, we used cell lines as well as the preclinical patient-derived mouse xenograft models obtained from the corresponding human tumor samples to determine these protein functions. We identified the CENPK–delta8 splice variant to be the most significant variant involved in the Abiraterone-resistance through gain- and loss-of-function experiments. Further, we examined the CENPK–delta8 specific binding partners and found FLOT1 and FLNA downstream. The FLOT1 is highly expressed in prostate tissue and its role reflects the cellular function of cytoskeleton, associated with cell proliferation [11,12,13,14,15,16,17,18], cell migration and tumor progression [12,19,20], cell invasion [16], and treatment resistance [11,19,21]. Similarly, FLNA functions as a scaffold for signaling molecules and closely links to cytoskeleton functions such as cell adhesion, cell movement, cell survival, the organization of extracellular matrix, metastasis, and chemoresistance [22,23,24,25]. Our study highlights the critical function of alternative splice variants in drug response and their potential to be prognostic biomarkers.

## 2. Materials and Methods

### 2.1. Cell Culture and Reagents

Human prostate cancer cell lines purchased from ATCC were routinely cultured in RPMI1640 medium (Gibco, Grand Island, NY, USA) supplemented with 10% fetal bovine serum (FBS) (Atlanta Biologicals, Flowery Branch, GA, USA) and 1% Pen–Strep (Gibco, Grand Island, NY, USA). All cell lines were characterized by STR analysis, tested to see if they were free from mycoplasma infection (IDEXX Laboratories, Westbrook, ME, USA), and cultured at 37 °C in a 5% CO_2_ incubator. Abiraterone acetate and pregnenolone were purchased from Selleckchem and dissolved in 100% ethanol or DMSO, respectively.

### 2.2. PDX Mouse Model Generation and RNA Sequencing

Tumor samples from patients were subjected to RNA-seq using methods indicated in a previous publication [10], followed by an analysis of ‘molecular signatures’ (e.g., splice variants) associated with response, or lack of response, to treatment followed by xenograft based functional studies.

In brief, pathology-confirmed metastatic tumor biopsy tissues of mCRPC were renal-capsule xenografted and a 25 mg testosterone pellet was subcutaneously implanted into 6–8 week old male CB17 NOD–SCID mice (Jackson Laboratories, Bar Harbor, ME, USA; Charles River Laboratories, Raleigh, NC, USA) and observed for at least 6 months or until the tumor reached 1.0–1.5 cm at maximal length, when sub-xenografts were expanded by subcutaneous implantation with harvested PDX tumor mixed with Matrigel (Corning Inc., New York, NY, USA) with no exogenous testosterone supplied. Early generation PDX tumors were collected for cryo-storage and next generation sequencing. In this study, pathology-confirmed PDX models generated from AA/P non-responder and responder bone metastatic tumor biopsy tissues were employed to either knockdown or overexpress splice variants and then test their function in Abiraterone drug resistance.

Body weight and tumor volumes (width × length) were measured two to three times per week with a digital caliper, and the average tumor volumes were determined. After the PDX tumor reached approximately 1000 mm^3^, mice were euthanized and the tumors were removed, dissected, and frozen at −80 °C for further analysis.

### 2.3. PDX 3D Organoid Generation

The PDX-derived organoid culture and cytotoxicity assay PDX tumor dissociation, tumor cell isolation, and organoid formation were described previously [26]. The tumor cell dissociation kit, cell strainer, and mouse cell depletion kit were purchased from Miltenyi Biotec. Harvested tumors were dissected into 2 mm sections and incubated with 5 mL of the human Tumor Dissociation enzyme mix. The tumor tissue was digested gently on a MACS Dissociator and passed through a 70 μm and a 40 μm MACS Smart Strainer, sequentially. After centrifugation and being washed with precooled washing buffer, mouse cells were removed by the Mouse Cell Depletion Purification Kit according to the manufacturer’s instructions. To culture a tumor organoid, 1.5–2 × 10^4^ live cells were cultured in 96 well NanoCulture Plate (Scivax Corp, Woburn, MA, USA) in 100 mL modified MEF medium (Phenyl-red free DMEM supplemented with 10% charcoal-stripped FBS, 1% glutamax, 1% sodium pyruvate, 1% nonessential amino acids, 1% penicillin–streptomycin (Life Technologies, Grand Island, NY, USA), supplemented with 5 mM Y27632 ROCK inhibitor (Tocris Bioscience, Bristol, UK) and 50 nM pregnenolone. The medium was carefully replaced every 3–5 days. Tumor organoids were allowed to grow for 2 to 5 days before drug testing. Organoids were then treated with the indicated concentration of Abiraterone before viability was examined by 3D celltiter kit (Promega, Madison, WI, USA). The solvent was used as the vehicle control.

### 2.4. Cytotoxicity Assay for Cells and 3D Organoids

Cells were seeded in phenol red-free RPMI (Gibco, Grand Island, NY, USA) medium with 10% charcoal-stripped FBS (Thermo Fisher Scientific, Waltham, MA, USA), and 50 nM pregnenolone was supplemented after 24 h. Next, cells were subjected to Abiraterone treatment at various doses for 3 days in 96-well plates and examined their viability by CyQUANT Kit (C7026, Thermo Fisher Scientific, Waltham, MA, USA). Independent experiments were repeated at least three times, with eight replicates for each experiment.

For 3D organoids, cells were seeded in Sphera Low-Attachment 96-well plates (174927, Thermo Scientific, Waltham, MA, USA) with 3D culture medium and treated at various doses 24 h later in 5 to approximately 8 replates (depending on the actual tumor size). Following treatment, viability was examined by CellTiter–Glo^®^ 3D Cell Viability Assay (G9683, Promega, Madison, WI, USA).

### 2.5. Clinical Data and Bioinformatic Analysis

For disease-free survival curve, high expression is compared to low expression (in TPM) in prostate cancer patients from TCGA database. For bioinformatic analysis, AA/P non-responder and responder mCRPC individual patient data were accessed via Mayo Clinic from the PROMOTE study.

### 2.6. Plasmids

For the disease-free survival curve, the Human *CENPK* splice variant and full-length *CENPK* were, respectively, cloned into the pIRES2–EGFP vector (Addgene, Watertown, NY, USA) along with inserts including Stag, Flag, and polylinker. The siRNAs of full-length *CENPK*, *FLNA*, and *FLOT1* were purchased from the predesigned SMARTpool (with a mixture of 4 siRNAs for a single gene) and the CENPK splice variant was customized (Horizon Discovery, Lafayette, LA, USA). Lipofectamine™ 2000 (11668030, Invitrogen, Waltham, MA, USA), RNAiMAX (13778075, Invitrogen, Waltham, MA, USA), and LTX with PLUS (15338100, Invitrogen, Waltham, MA, USA) transfection reagents were ordered from Thermo Fisher Scientific for knockdown and overexpression experiments. Cells were temporarily transfected with either Flag–GFP–CENPK–delta8 construct, Flag–GFP–CENPK construct, or *CENPK*, *FLNA*, *FLOT1* siRNA For stable 22Rv1 Δ8CCENPK OE cell line, cells were infected by lentivirus generated in HEK–293T. The lentivirus supernatant was concentrated using the Takara Lenti–X Concentrator and were then added to the 22Rv1 cell line along with 10 μg/mL polybrene for 72 h followed by G418 selection. 22Rv1 with no insert following same G418 treatment was used as control for selection. Transfection efficiency of CENPK–delta8 and CENPK in cells was monitored by using EVOS Floid Imaging System (Thermo Fisher Scientific) to visualize the GFP signal. The sequences of full-length CENPK, CENPK–delta8, and primers are listed in Supplementary Table S1. Knockdown and overexpression were validated via qRT–PCR (Figures S5–S7).

### 2.7. Co-Immunoprecipitaion (Co–IP) and Mass Spectrometry

The Co–IP assays were performed with primary antibody against the target protein, followed by mass spectrometric (MS) analysis of the co-precipitated proteins, as we previously reported [27]. The Co–IP was performed by using the Pierce™ Classic Magnetic IP/Co–IP Kit (88804, Thermo Fisher Scientific, Waltham, MA, USA) according to manufacturer’s instructions. In brief, cells were treated with Abiraterone, washed twice with ice-cold PBS, scraped down and centrifuged. Harvested cell pellets were lysed in ice-cold IP Lysis buffer (pH 7.4, 0.025 M Tris, 0.15M NaCl, 0.001 M EDTA, 1% NP40, 5% glycerol) containing Halt™ protease and phosphatase inhibitor cocktail (78444, Thermo Fisher Scientific, Waltham, MA, USA) for 5 min and then centrifuged at approximately 13,000× *g* for 10 min to pellet the cell debris. After quantifying protein concentration by using the BCA Protein Assay Kit (23227, Thermo Fisher Scientific), the samples were incubated with Flag primary antibody (our GFP–CENPK–delta8 and GFP–CENPK vectors have Flag tag) at 4 °C overnight. The next day, after incubation with Pierce™ Protein A/G Magnetic Beads for 2 h at 4 °C, the antigen sample/antibody mixture was collected by a magnetic stand and washed 3 times with fresh IP Wash Buffer. The samples with Flag–CENPK–delta8 or Flag–CENPK complex were then eluted by heating at 100 °C for 5 min in Laemmli buffer. Next, the complex was loaded onto Mini–PROTEAN^®^ TGX™ Precast Protein Gels (Bio-Rad, Hercules, CA, USA) for protein separation. The gels were stained by Pierce™ Silver Stain (24600, Thermo Fisher Scientific) and then interested bands were cut for MS analysis. The MS and results analysis were performed by the Taplin Biological Mass Spectrometry Facility at Harvard Medical School (Boston, MA, USA). Two rounds of Co–IP were performed. Two tags in the vector were used for Co–IP. Protein lysate that had been transfected with empty vector was used as control. The MS results were validated by Western blot. Pathway analysis of IP–MS data was performed by Erichr portal (https://maayanlab.cloud/Enrichr/) (accessed on 1 March 2022) [28,29]. The CENPK splice variant and wild-type CENPK binding partners determined by IP–MS are listed in Supplementary Table S2.

### 2.8. Transwell Migration and Invasion Assays

For the Transwell migration assay, cells were engineered (either knockdown or overexpression target element) and placed in the inserts (onto the permeable membrane with pore size of 8.0 μm) of 24-well plates (Corning, NY, USA) in serum-free RPMI 1640 medium supplemented with 1% Pen–Strep. The 10% FBS with the same medium was added to the lower compartment as a chemoattractant. Cells that migrated to the lower surface of the membrane were fixed with methanol for 20 min and stained with crystal violet for 30 min. Cell images were captured by Invitrogen EVOS microscope. Cell numbers were analyzed using the Fiji software 2.9.0. For the invasion assay, the same procedures were followed, but the plates were pre-coated with thin layer of Matrigel by the manufacturer (Corning, NY, USA) and stored at −20 °C.

### 2.9. Wound Healing Assay

Cells were engineered and grown to 100% confluence in 6-well plates. Forty-eight hours before wound scratch, RPMI 1640 medium containing 10% FBS and 1% Pen–Strep was replaced by phenol red-free RPMI medium (Gibco, Grand Island, NY, USA) with 10% charcoal stripped FBS. The 50 nM pregnenolone was supplemented after 24 h. Wounds were scratched in the monolayer with 100 μL pipette tips. Suspended cells and debris were washed out with warm culture media. Cells were subjected to Abiraterone treatment at 5 μM and/or 10 μM. The migration of cells into the wounded areas was imaged at the indicated times using an Invitrogen EVOS microscope (Thermo Fisher Scientific) and quantified by Fiji software with the wound healing tool added, an enhanced version of ImageJ 1.53p. At least three individual experiments were repeated. Three wells in each experiment were measured for the distance of the migrating cells to the origin of the wound.

### 2.10. Colony Formation Assay

Cells were seeded into 6-well plates and treated with Abiraterone in duplicate following the same procedures as indicated above. Cells were then allowed to grow until visible colonies formed with medium renewed every 7 days. Colonies were fixed with methanol for 20 min, stained with crystal violet for 30 min, and then washed with ddH2O until the background was clear. ImageJ with ColonyArea plugin was used for data analysis [30].

### 2.11. qRT–PCR

Total RNA for qRT–PCR was extracted from cell lines using the Quick-RNA MiniPrep Kit (Zymo Research, Irvine, CA, USA) according to the manufacturer’s protocol. The qRT–PCR was performed using the Power SYBR^®^ Green RNA-to-CT 1-Step Kit (Life Technologies, Grand Island, NY, USA) on the Applied Biosystems™ QuantStudio™ real-time PCR system (Thermo Fisher Scientific) [31]. The qPCR primers were ordered from either QuantiTect^®^ (QIAGEN, Germantown, MD, USA) or IDT Inc. (Coralville, IA, USA). Gene expression analyses were performed using the ΔΔCt method, and GAPDH was used as the housekeeping gene for internal reference. Independent experiments were repeated at least three times. Primer sequences are listed in Supplementary Table S1. To profile CENPK splice variant in samples, primers were designed to specifically span the Exon 7 and Exon 9 junction [32].

### 2.12. Western Blot Analysis and Immunofluorescence Staining

Following Abiraterone/vehicle treatment and ice-cold PBS wash, cells from early passage were harvested and lysed by homogenizing and sonicating in RIPA buffer with Halt™ protease and phosphatase inhibitor cocktail, and then centrifuging at 12,000× *g* for 10 min. Proteins were quantified and normalized using the BCA Protein Assay Kit. Equal quantities of denatured protein were loaded onto the Mini-PROTEAN^®^ TGX™ Precast Protein Gels which were then blocked with 5% non-fat milk at room temperature for 1 h. After washing with TBST, membranes were incubated with primary antibody in TBST supplemented with 1% BSA at 4 °C overnight. Following incubation, membranes were washed vigorously five times in TBST wash buffer and then incubated with horseradish peroxidase (HRP)-labelled secondary antibody in 5% non-fat milk at room temperature for 1 h. The SuperSignal™ West Femto Maximum Sensitivity Substrate (34095, Thermo Fisher Scientific, Waltham, MA, USA), an ultra-sensitive enhanced chemiluminescent substrate, was applied to the membranes, and radiographic images were captured by use of the ChemiDoc™ Touch Image System (Bio-Rad, Hercules, CA, USA). The GAPDH protein was employed as a loading control.

For immunofluorescence staining (IF), cells were grown in a 16-well CultureWell™ Chambered Coverglass (C37000, Thermo Fisher Scientific). After treatment with either vehicle or Abiraterone, cells were fixed by 4% (*w*/*v*) methanol-free formaldehyde (28906, Thermo Fisher Scientific) in PBS at room temperature for 20 min, followed by triple wash with ice-cold PBS. Then, cells were blocked and permeabilized with 3% BSA and 0.1% Triton X-100 in PBS for 1 h. After that, cells were incubated in primary antibodies at 4 °C overnight. The next day, cells were washed by PBS and incubated in Alexa dye-conjugated secondary antibody. With triple PBS wash, cells were mounted by mounting medium with DAPI. The visualization of cells with nucleus in blue was captured by confocal microscopy (Zeiss LSM 780, Oberkochen, Germany). The 2D and 3D image data were analyzed by both automated ZEN microscopy software 3.5 and Fiji software as previously published [31,33].

Antibodies against various proteins were as follows: Flag (F3165, Millipore Sigma, Burlington, VT, USA, 1:1000), FLNA for Western blot (#4762, Cell Signaling, Danvers, MA, USA, 1:1000), FLNA for immunofluorescent staining (ab76289, Abcam, Waltham, MA, USA, 1:100), FLOT1 (#18634, Cell Signaling, 1:1000 for Western blot or 1:50 for immunofluorescent staining), GAPDH (5174S, Cell Signaling, 1:1000), anti-mouse IgG, HRP-linked antibody (7076S, Cell Signaling, 1:2000), anti-rabbit IgG, HRP-linked antibody (7074S, Cell Signaling, 1:2000), anti-rabbit IgG (H+L), F(ab′)2 Fragment (Alexa Fluor 488 conjugate) antibody (#4412, Cell Signaling, 1:2000), anti-rabbit IgG (H+L), F(ab′)2 Fragment (Alexa Fluor 594 conjugate) antibody (#8889, Cell Signaling, 1:2000), anti-mouse IgG (H+L), F(ab′)2 Fragment (Alexa Fluor 488 conjugate) antibody (#4408, Cell Signaling, 1:2000), Extracellular Matrix Dynamics Antibody Sampler kit (#63387, Cell Signaling, 1:1000).

### 2.13. Flow Cytometry

For cell apoptosis analysis, cells were knocked down and then subjected to acute Abiraterone treatment. Later, cells were washed, trypsinized, and resuspended in PBS containing 5% FBS and put on ice. Cells were incubated with reagent containing Annexin V and propidium iodide (V13241, Thermo Fisher Scientific) for 15 min at room temperature, then analyzed using flow cytometry (BD FACS CantoX, Bridgewater, NJ, USA) with the Mayo Core Facility cell-cycle template. All staining was performed in the darkness. Gates were set based on technique controls in each experiment. FlowJo software v10.9 (FlowJo, LLC, Ashland, OR, USA) was used for data processing.

### 2.14. Statistical Analysis

Statistical calculations were performed using GraphPad Prism 8.0 (San Diego, CA, USA) and R 4.1.2 software. Normally distributed raw data were analyzed with either Student’s *t*-test (2-group comparisons) or one-way ANOVA (>2-group comparisons) with Tukey post hoc test unless otherwise mentioned. Survival curves were plotted by the Kaplan–Meier method and compared using the log–rank test. Statistically significant differences were denoted as *, *p* < 0.05; **, *p* < 0.01; and ***, *p* < 0.001.

## 3. Results

### 3.1. Splice Variants Are Associated with Abiraterone Response

We previously identified splice variants that were associated with AA/P response in mCRPC patients using the RNA–Seq of samples collected from the bone metastasis from patients enrolled in the Mayo Clinic PROMOTE study [10]. The identified splice variant genes (*CENPK*, *PKN1*, *KIAA1324*, *EMD*, and *TSC2*) associated with response were mainly related to mitosis, PI3K–Akt cell survival/proliferation signaling, and tumor progression and metastasis (Figure S1A). To further characterize the significance of these splice variants in AA/P resistance, the in vitro function of these splice variants was investigated in cell lines and 3D PDX tumor organoids derived from the PROMOTE responders and non-responders. These variants were expressed to various degrees in our PDX derived organoids and commonly used prostate cancer cell lines (Figure S1B,C). The expression patten in different organoids matched with the results from corresponding clinical tumors. Among these splice variants, CENPK, PKN1, and KIAA were upregulated, while EMD and TSC2 were downregulated in the non-responders.

**Figure 1:**
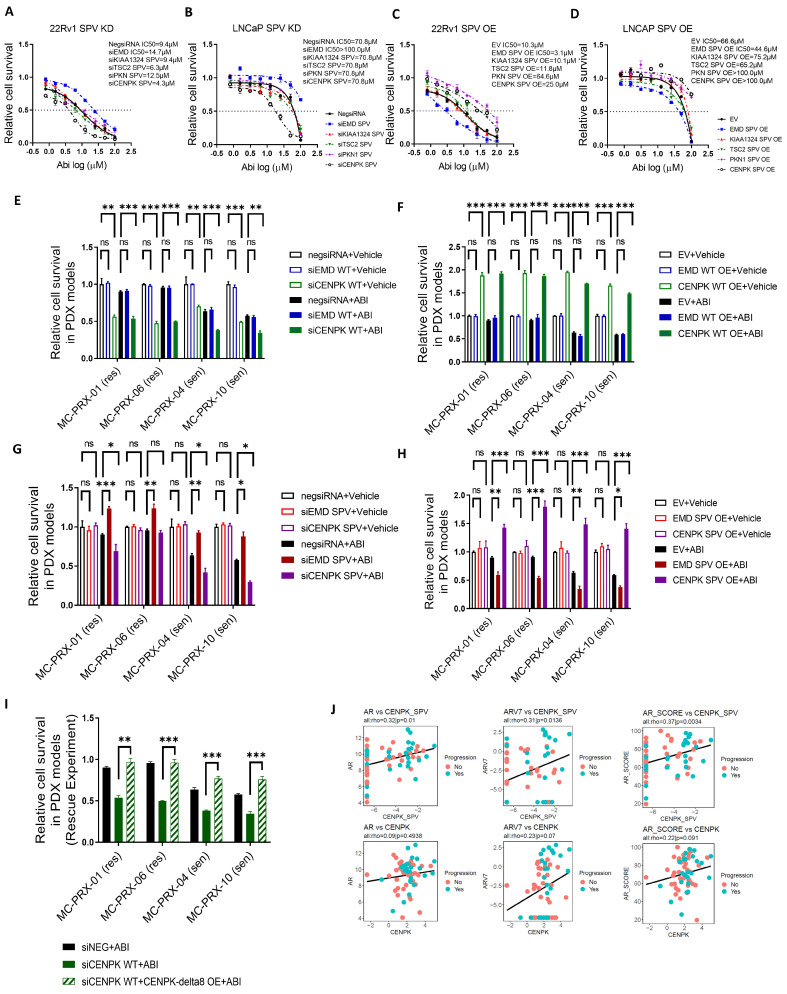
Splice variants in prostate cancer cells and PDX 3D organoids in response to Abiraterone. Cytotoxicity assays of knockdown of splice variants in 22Rv1 (**A**) and LNCaP (**B**) cells treated with various doses of Abiraterone are shown. These genes were obtained from RNA-Seq data including *EMD*, *KIAA*, *TSC2*, *PKN1*, and *CENPK*. Cytotoxicity assays of splice variant overexpression in 22Rv1 (**C**) and LNCaP (**D**) cells treated with various doses of Abiraterone are shown. Cytotoxicity assays of PDX organoids (**E**–**H**) treated with indicated doses of Abiraterone or vehicle are shown. The 3D organoids were (**E**) knocked down with either wild-type EMD or wild-type CENPK, (**F**) overexpressed with wild-type EMD or wild-type CENPK, (**G**) knocked down with either EMD-cryptic 5′SS or CENPK–delta8, or (**H**) overexpressed with either EMD-cryptic 5′SS or CENPK–delta8. (**I**) Cytotoxicity assays of 3D organoids following Abiraterone treatment. The organoids were either with wild-type CENPK knockdown or with wild-type CENPK knockdown plus CENPK–delta8 overexpression. The PDX models come from two responder patients (PR06 and PR12) and two non-responder patients (PR03 and PR08). PR06: MC-PRX-04; PR12: MC-PRX-10; PR03: MC-PRX-01; PR08: MC-PRX-06. The *X*-axis indicates concentrations of Abiraterone. All data were presented as mean ± S.E.M. of at least five replicates and normalized to vehicle treatment (as shown on the right side). (**J**) Correlation plots of wild-type CENPK, CENPK–delta8, androgen receptor (AR), and androgen receptor splice variant-7 (AR–V7), as well as AR score plots with CENPK wild type and delta8. Patient data were from Mayo Clinic PROMOTE cohort. WT, wild-type CENPK; SPV, CENPK splice variant; OE, overexpression; KD, knockdown. Data were presented as mean ± S.E.M. of two replicates. Neg siRNA, nontarget siRNA control; EV, empty vector; WT, wild type; SPV, splice variant; OE, overexpression; ABI, Abiraterone. Statistically significant differences were denoted as *, *p* < 0.05; **, *p* < 0.01; ***, *p* < 0.001; ns, not significant.

We first determined cell proliferation after knockdown of each gene (refer to the Supplementary Table S1 for siRNA sequence), respectively, in prostate cancer cell lines, 22Rv1 and LNCaP, both of which are reported to be at least partially androgen-dependent and express AR, the primary target of Abiraterone, CYP17A1 [34,35,36,37]. Compared to PKN1, KIAA1324, and TSC2 splice variants, CENPK–delta8 and EMD-cryptic 5′SS showed the most significant changes in Abiraterone cytotoxicity (Figure 1). Therefore, we further focused on CENPK–delta8 and EMD-cryptic 5′SS. Firstly, we knocked down the identified splice variant genes (*CENPK*, *PKN1*, *KIAA1324*, *EMD*, and *TSC2*) in 22Rv1 (Figure 1A) and LNCaP (Figure 1B), then we overexpressed these variants in 22Rv1 (Figure 1C) and LNCaP (Figure 1D). Cell proliferation in the presence of increasing Abiraterone concentrations (the same drug utilized in PROMOTE study) showed that knockdown (KD) of CENPK–delta8 (with the skipping of Exon 8) resulted in a significant decrease in cell proliferation (Figure 1A,B), while CENPK–delta8 overexpression (OE) showed the opposite results, increasing cell proliferation (Figure 1C,D). Following the knockdown of EMD-cryptic 5′SS, the downregulated splice variant in non-responders led to a significantly increased Abiraterone response while a decreased response upon EMD-cryptic 5′SS overexpression was observed.

All these results were consistent with the clinical association results in that CENPK–delta8 expression was high in Abiraterone-resistant mCRPC patients and EMD-cryptic 5′SS expression was low in Abiraterone non-responders vs. responders.

### 3.2. CENPK–delta8 as an Abiraterone-Resistant Splice Variant Biomarker in mCRPC Patients

We further tested the effect of the two variants, CENPK–delta8 and EMD-cryptic 5′SS, both of which are involved in mitosis in response to Abiraterone in 3D organoids derived from PDX models. The models came from tumor biopsies obtained from responders (MC-PRX-04, MC-PRX-10, sensitive to AA/P treatment) and non-responders (MC-PRX-01 and MC-PRX-06, resistant to AA/P treatment) in the PROMOTE study. Responders and non-responders were clinically categorized by the Mayo Clinic, based on the study criteria (NCT #01953640). Patient tumor cells obtained from these PDX models were grown into 3D organoids. Cell proliferation was measured in wild-type or CENPK–delta8 KD or OE organoids after five days of DMSO or Abiraterone treatment (Figure 1E–H). The loss of wild-type CENPK decreased cell proliferation, while overexpression increased cell proliferation. Additional Abiraterone did not change organoid survival significantly compared to KD or OE organoids treated with DMSO (Figure 1E,F). As expected, in Abi-sensitive organoids, Abiraterone treatment showed a better inhibition of proliferation than that in Abi-resistant organoids. However, although CENPK–delta8 KD or OE did not significantly change survival, the addition of Abiraterone treatment further reduced organoid survival in CENPK–delta8 KD organoids, and the reduction was more prominent in Abiraterone sensitive organoids. However, in the CENPK–delta8 OE organoid, treatment of Abiraterone significantly increased organoid proliferation compared with parental organoids treated with Abiraterone. Similar experiments were conducted with EMD. The KD or OE of WT EMD did not change the organoid proliferation and Abiraterone response (Figure 1E,F). Similarly, the OE or KD EMD splice variant did not significantly affect organoid proliferation, but wild-type CENPK knockdown, in the absence of treatment, led to decreased cell viability. Wild-type CENPK and CENPK–delta8 coexist in tumors, and both contribute to cell survival and progression, as recently reported [38,39]. While parental organoids treated with Abiraterone decreased the proliferation, and the effect is more significant in sensitive organoids, KD of the EMD variant increased organoid proliferation in the presence of Abiraterone compared with parental organoids treated with the drug; OE showed the opposite effect (Figure 1G,H). Although CENPK–delta8 knockdown in MC-PRX-06 had minimal effects due to low transfection efficiency, the rescue experiment in Figure 1I demonstrates the proliferative effect of CENPK variant in Abiraterone response.

To further validate the potential clinical impact of CENPK–delta8, we used prostate cancer patient data from both TCGA and TCGA SpliceSeq. The CENPK high expression cohort is significantly associated with poor prognosis (Figure S1D). The CENPK–delta8 showed a high expression in medium/high-grade prostate cancer with a Gleason score of 7–9 (Figure S1E). Since AR/AR–V7 and AR scores are associated with prostate cancer progression, we analyzed our PROMOTE study to derive AR and AR–V7 levels as well as AR activity score [40]. We then determined whether CENPK–delta8 might correlate with AR/AR–V7 and AR activity scores. In Figure 1J, CENPK–delta8 positively correlated with the expression of AR and AR–V7. High CENPK–delta8 expression also correlated with a high AR activity score in our PROMOTE study, consistent with its potential role in cell proliferation and resistance to Abiraterone.

**Figure 2:**
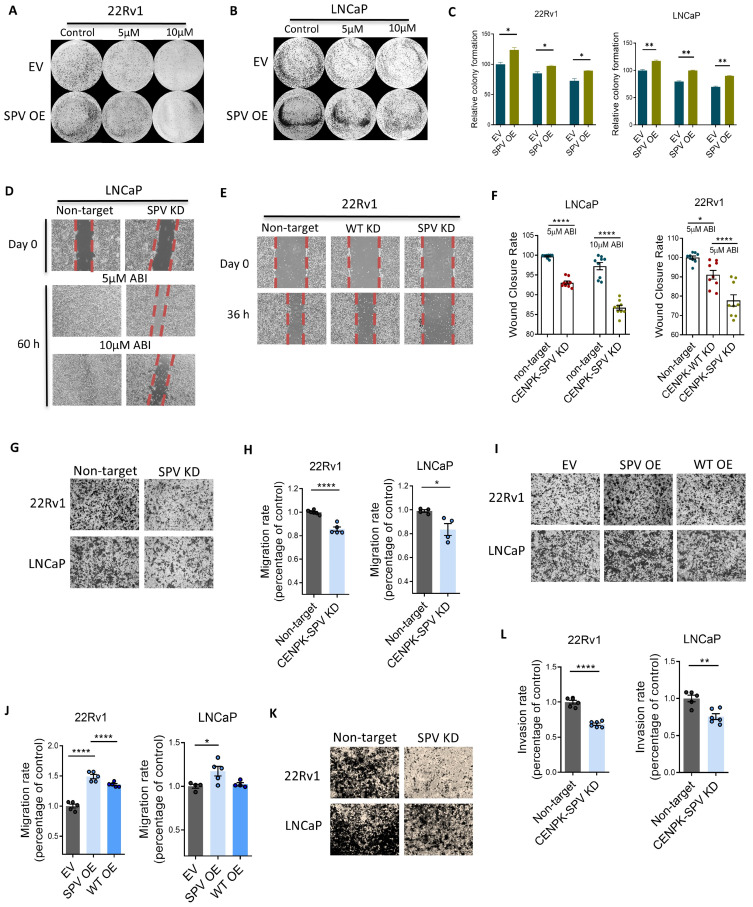
CENPK splice variant promoting cell migration and invasion. (**A**,**B**) Colony formation assays of CENPK–delta8 OE 22Rv1 and CENPK–delta8 OE LNCaP upon indicated Abiraterone treatment and their corresponding data analysis (**C**). The dashed/solid lines are the boundary between cells and empty space. All patient-derived organoids in this study have been examined for pathology validation before experiments. Data were presented as mean ± S.E.M. of different areas in two to three replicates. Representative images of wound healing assays in (**D**) LNCaP (upon 5 μM and 10 μM Abiraterone treatments) and (**E**) 22Rv1 (upon 5 μM Abiraterone treatment) with their corresponding quantification (**F**). Data were presented as mean ± S.E.M. of different areas in three replicates. Representative images of transwell migration assays in (**G**) CENPK–delta8–KD 22Rv1 and LNCaP and the corresponding data analysis (**H**). Representative images (**I**) of transwell migration assays in 22Rv1 and LNCaP with the overexpression of either empty vector, wild-type CENPK, or CENPK–delta8, and their corresponding data analysis (**J**). Representative images (**K**) of transwell invasion assays in CENPK–delta8–KD 22Rv1 and LNCaP, and the corresponding data analysis (**L**). Cells were treated with 5 μM Abiraterone. Migration and invasion imaging was completed using a 10× objective. EV, empty vector; SPV, CENPK splice variant or CENPK–delta8; WT, wild-type CENPK; OE, overexpression; KD, knockdown. Statistical significance indicated unpaired *t*-test: * *p* < 0.05, ** *p* < 0.01, **** *p* < 0.0001.

Because the proliferative effect of CENPK variant on Abiraterone response was much more significant, we decided to further pursue the mechanisms underlying CENPK splice variant effect in Abiraterone response. With a focus on CENPK function, we then knocked down endogenous wild-type CENPK and simultaneously overexpressed CENPK–delta8. Following Abiraterone treatment, the loss of wild-type CENPK decreased cell proliferation, while overexpression of CENPK–delta8 restored cell proliferation in 3D organoids (Figure 1I). Furthermore, overexpression of CENPK–delta8 in 22Rv1 and LNCaP cells significantly increased the number of colonies (Figure 2A–C). The data suggest that the CENPK–delta8 variant promotes prostate cancer growth and plays an important role in Abiraterone resistance.

Collectively, this evidence confirms the critical role of CENPK–delta8 in Abiraterone drug resistance, identifying CENPK–delta8 as a significant Abiraterone-resistant splice variant biomarker.

### 3.3. CENPK–delta8 Contribution to Abiraterone-Resistant Phenotype through Enhanced Cell Migration

We examined whether CENPK–delta8 might affect cell migration. Indeed, KD CENPK–delta8 showed decreased migration upon Abiraterone treatment compared to nontarget control (Figure 2D–F). These data were validated by transwell migration and invasion assays with knockdown and overexpression of CENPK–delta8 in LNCaP and 22Rv1 cells (Figure 2G–L). Migrated/invaded cells were stained with crystal violet. Taken together, they increased CENPK–delta8 enhanced cell migration and cell proliferation.

**Figure 3:**
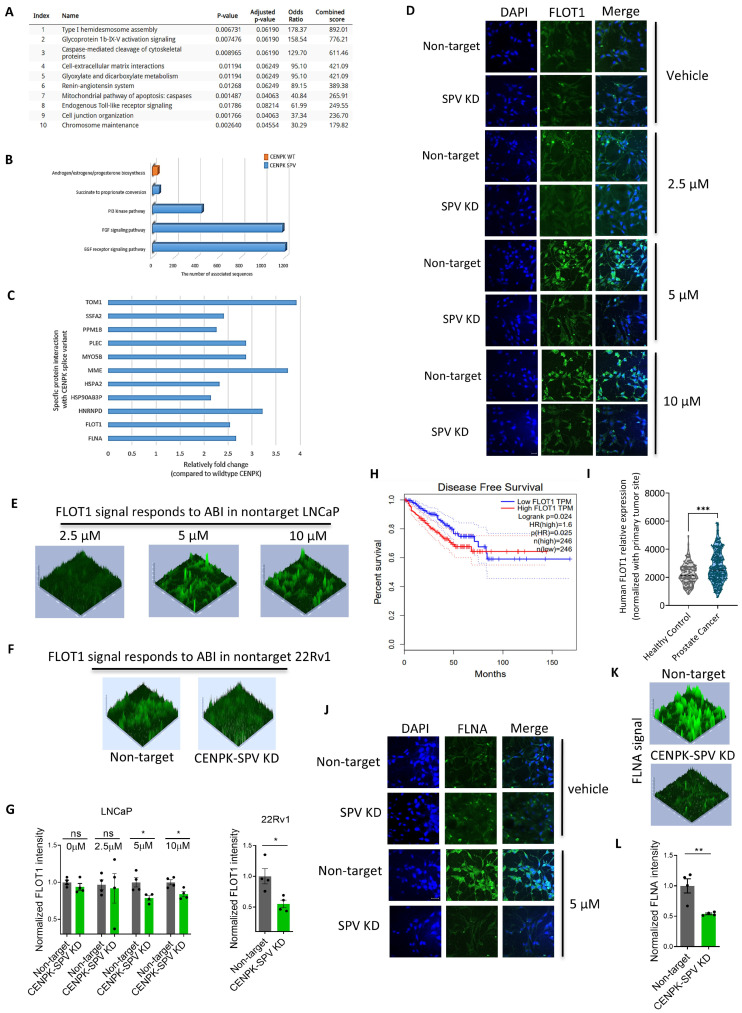
Pathways and proteins specifically binding CENPK–delta8 in Abiraterone response. (**A**) Unique binding partners with CENPK–delta8 in CENPK SPV–OE LNCaP cells of IP–MS signals, compared to wild-type CENPK overexpression. (**B**) In comparison, unique pathways in CENPK WT–OE (in orange) and CENPK SPV–OE (in blue) LNCaP cells, respectively. (**C**) Predicted function analysis in CENPK SPV–OE through enriched pathways using the BioPlanet 2019 database set of Enrichr (see methods). CENPK SPV, CENPK–delta8; SPV, splice variant; WT, wild type. Representative confocal images of immunofluorescent staining with FLOT1 in CENPK SPV–KD LNCaP cells treated with various doses of Abiraterone (**D**). Scale bar, 50 μm. Representative 3D plots of FLOT1 intensity signal in nontarget/wildtype LNCaP with three different doses of Abiraterone (**E**) and 22Rv1 with 5 μM Abiraterone (**F**). (**G**) FLOT1 fluorescent intensity quantification in nontarget/wildtype LNCaP and 22Rv1. (**H**) Disease-free survival analysis in FLOT1 high expression vs. low expression (in TPM) prostate cancer patients based on TCGA data. The dashed lines show the confidence interval. (**I**) *FLOT1* relative expression in healthy subjects and prostate cancer patients based on GEO database. Representative confocal images (**J**) of immunofluorescent staining with FLNA in CENPK SPV–KD LNCaP cells treated with vehicle and 5 μM Abiraterone, 3D intensity signal (**K**), and FLNA intensity analysis (**L**). Scale bar, 50 μm. Data were presented as mean ± S.E.M. of four replicates. Scale bar: 100 μm. Statistical significance indicated unpaired *t*-test: * *p* < 0.05, ** *p* < 0.01, *** *p* < 0.001; ns, not significant. The 3D plots were analyzed in the same setting. SPV, splice variant; WT, wild type. ABI, Abiraterone; KD, knockdown.

To further explore the CENPK–delta8 function, we performed immunoprecipitation using the tag antibodies followed by mass spectrometry (IP–MS) experiments in CENPK–delta8 OE and wild-type CENPK OE LNCaP cells, respectively (refer to the Supplementary Table S2; 3 groups in total, with empty vector in the control group). Our CENPK WT and CENPK–delta8 vectors were designed to have two tags—GFP and DYKDDDK flag. The results from IP–MS were validated by Western blot experiments (Figure S2). The CENPK is a subunit of CENP centromeric complex consisting of CENPH and CENPI. In this study, we did not obtain the CENPK–delta8 specific binding antibody. Therefore, CENPH and CENPI antibodies were used to confirm the success of the CENPK–delta8 pulldown instead. Because the CENPK–delta8 vector also expresses the FLAG tag, the FLAG antibody was also used for further validation in Western blot. The pathway analysis using all top identified interacting proteins indicated that these genes are enriched in the cytoskeleton/migration (i.e., caspase-mediated cleavage of cytoskeletal proteins, cell-extracellular matrix interactions, cell junction organization), mitochondria-associated apoptosis, and mitosis (i.e., chromosome maintenance) (Figure 3A). Compared to CENPK–delta8, the unique protein binding partners of wild-type CENPK in WT OE cells are involved in the pathway of androgen/estrogen/progesterone biosynthesis (Figure 3B). In comparison, the unique interacting genes with CENPK–delta8 were enriched in cell proliferation and migration/metastasis signaling (i.e., succinate to propionate conversion, PI3, FGF, and EGF receptor signaling) [41,42,43,44,45] (Figure 3B). The proteins specifically interacting with CENPK–delta8 are listed in Figure 3C.

Among the positive binding partners in IP–MS (Figure 3), FLOT1 and FLNA were closely related to the cytoskeleton, metastasis (migration and invasion), and drug resistance [21,22,46,47]. The FLOT1 has been implicated as being associated with a poor prognosis in breast cancer [12,48,49], colon cancer [18], esophageal squamous cell carcinoma cells [15], and many other cancers [20,50,51,52]. We observed the effect of CENPK variant on cell migration and invasion. Therefore, we further studied the functions of FLOT1 and FLNA in the context of CENPK–delta8. We first performed immunochemistry to examine the role of FLOT1 in Abiraterone response. The FLOT1 expression was upregulated in the presence of increasing concentrations of Abiraterone in LNCaP (Figure 3D,E). However, KD CENPK–delta8 in LNCaP and 22Rv1 cells significantly decreased the FLOT1 level induced by Abiraterone (Figure 3D–F). Furthermore, the disease-free survival curve showed that higher FLOT1 expression in prostate cancer tumor patient cohorts is significantly associated with poor prognosis (Figure 3G). The GEO data show prostate cancer patients have significantly higher expression than healthy subjects (Figure 3H).

Next, we specifically investigate the role of FLNA, which belongs to the cell-extracellular matrix interactions pathway. Similarly, the immunofluorescent images showed that FLNA expression was upregulated with increasing doses of Abiraterone, while KD CENPK–delta8 in LNCaP cells downregulated FLNA expression induced by Abiraterone (Figure 3I–K).

**Figure 4:**
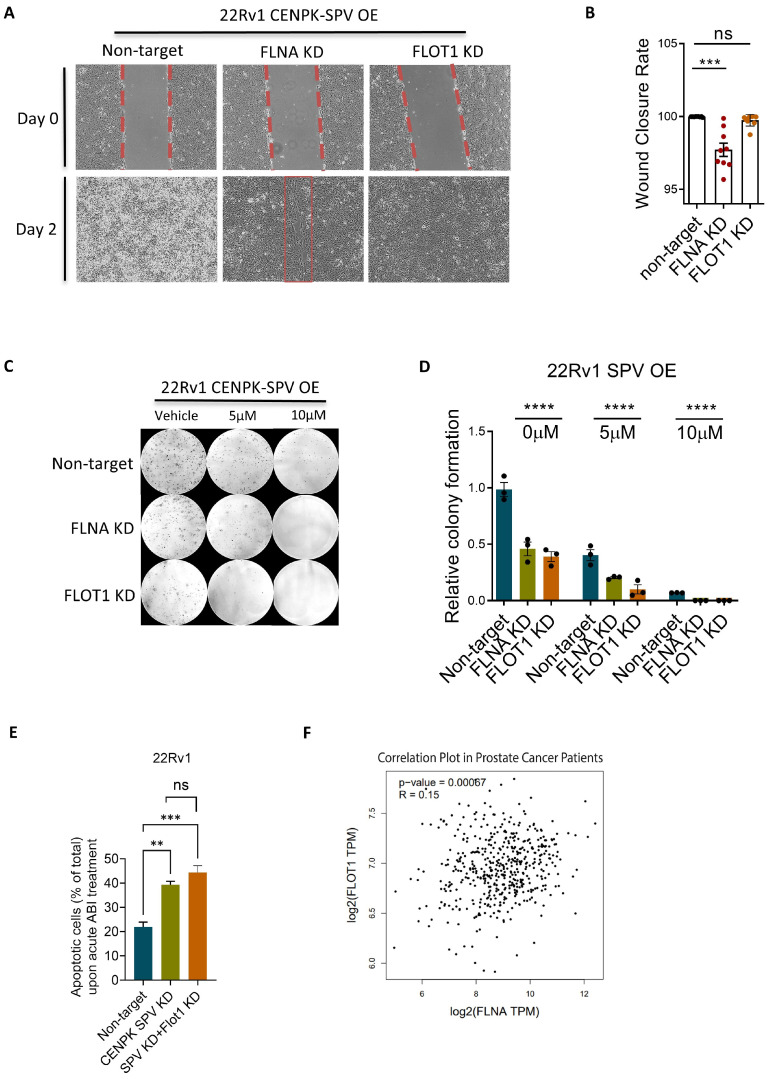
The functional analysis of FLNA and FLOT1 knockdown in CENPK–SPV overexpressed cells. (**A**) Wound healing assays of FLNA KD and FLOT1 KD in CENPK–delta8–OE 22Rv1 cells treated with 5 µM Abiraterone and the corresponding data analysis (**B**). Migration and invasion imaging was completed using a 10× objective. The dashed/solid lines are the boundary between cells and empty space. Data were presented as mean ± S.E.M. of eight replicates and normalized to vehicle treatment (as shown on the right side ‘control_non-target’). Colony formation assays of FLNA KD and FLOT1 KD in CENPK–delta8–OE 22Rv1 cells treated with indicated doses of Abiraterone (**C**) and the corresponding data analysis (**D**). (**E**) Annexin V/PI staining flow cytometry results in 22Rv1 following Abiraterone acute treatment. Cells had either CENPK–delta8 KD or CENPK–delta8 and FLOT1 double KD. (**F**) The correlation analysis between FLOT1 and FLNA expression (in TPM) in prostate cancer patients is based on TCGA data. Data were presented as mean ± S.E.M. of two replicates. Statistical significance indicated unpaired *t*-test: ns, not significant, ** *p* < 0.01, *** *p* < 0.001, **** *p* < 0.0001; ns, not significant. SPV, CENPK splice variant or CENPK–delta8; OE, overexpression; KD, knockdown.

We further studied how FLNA and FLOT1 might contribute to Abiraterone-resistant phenotype by overexpressing CENPK–delta8 and simultaneously knocking down either *FLNA* or *FLOT1* in 22Rv1 cells. We then determined cell migration in CENPK–delta8 OE and FLNA KD cells using the wound healing assay. The results showed that OE CENPK–delta8 significantly increased cell survival (Figure S3) compared with control cells of empty vector, while addition of KD FLNA reduced the migration rate induced by OE CENPK–delta8 and addition of KD FLOT1 decreased cell growth (lower cell density), indicating that the CENPK–delta8 effect on cell migration might be through FLNA and/or FLOT1 (Figure 4A,B). We then further tested the effect of CENPK–delta8 OE and FLNA/FLOT1 KD on cell proliferation assessed by colony formation. The colony formation analysis confirmed the results that FLNA KD and FLOT1 KD significantly reduced the number of colonies that was induced by OE CENPK–delta8 in 22Rv1 (Figure 4C,D). The annexin V/PI staining flow cytometry results showed that Abiraterone induced greater cell apoptosis in CENPK–delta8 knockdown cells compared to controls, but there was no significant difference between the CENPK–delta8 knockdown cells and those with double knockdown of CENPK–delta8 and FLOT1 (Figure 4E). These results confirm that Abiraterone’s apoptotic effect is mediated through CENPK–delta8, with FLOT1 potentially interacting with CENPK–delta8 to contribute to this effect. Based on TCGA data, FLOT1 expression is associated with FLNA expression in prostate cancer (Figure 4F), consistent with the evidence above. These results suggest the role of FLNA and FLOT1 in CENPK–delta8 regulated pathways that affect cell migration and proliferation, contributing to Abiraterone resistance.

Taken together, these results implicate an important role for CENPK–delta8-associated FLNA and FLOT1 signaling in mCRPC and Abiraterone response.

### 3.4. CENPK–delta8 Is Associated with Known Clinically Relevant Mutations

**Figure 5:**
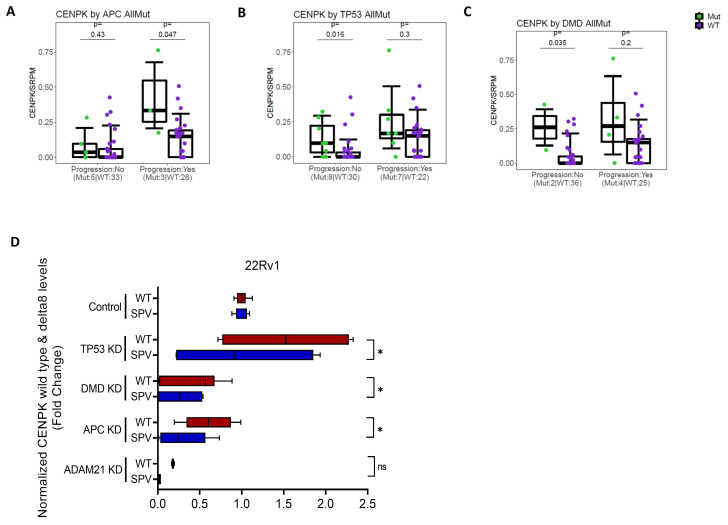
Knockdown clinically relevant genes identified from Mayo PROMOTE patients reducing CENPK splice variant expression. (**A**–**C**) Bioinformatic analysis of CENPK–delta8 by mutation in relevant cancer genes, based on the sequencing data of the PROMOTE clinical study at Mayo Clinic. The qRT–PCR validation with Abiraterone treatment (**D**) in 22Rv1 after KD of the relevant genes. Statistically significant differences were denoted as *, *p* < 0.05; ns, not significant.

To inform future diagnosis, prognosis, and treatment choice in mCRPC patients, we investigated if there are any known clinically relevant cancer mutations associated with CENPK–delta8. Bioinformatic analysis of mCRPC patient database from the PROMOTE study at Mayo Clinic revealed that CENPK–delta8 is significantly associated with APC mutation in Abiraterone progression setting (Figure 5A). In addition, we found that TP53 and DMD mutations as well as the ADAM21 mutation were also significantly related to CENPK–delta8, (Figure 5B,C and Figure S4). Then, we knocked down these genes and checked expression levels of CENPK splice variant and wild type. The qRT–PCR validation in 22Rv1 cells following vehicle and Abiraterone treatment showed that the knockdown of APC, TP53, and DMD significantly decreased CENPK splice variant gene expression compared to wild type (Figure 5D). Taken together, loss of function mutations in these APC, TP53, and DMD genes may result in downregulation of CENPK–delta8, leading to decreased cell proliferation and migration.

## 4. Discussion

**Figure 6:**
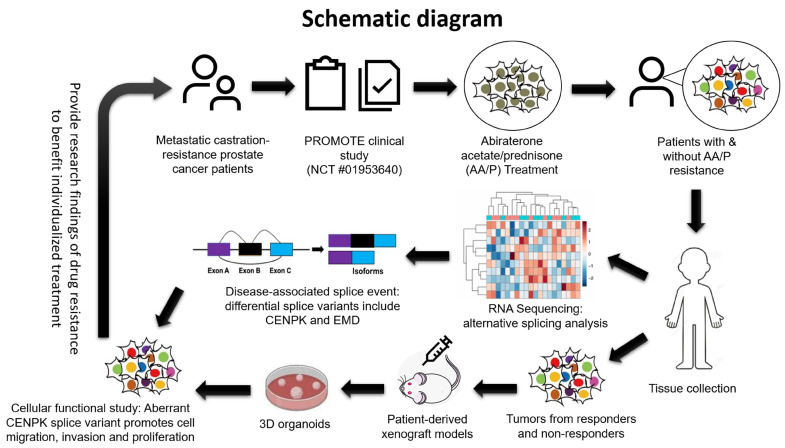
The schematic diagram for the functional study of splice variants in mCRPCs. Patients were recruited in the PROMOTE clinical trial to identify molecular signatures with response or lack of response to the standard treatment AA/P. Tumor tissues were employed to perform RNA–Seq (this is a cartoon, not data) for the analysis of disease-associated alternative splicing events and to generate PDX mouse models for the source of 3D organoids. The function of the CENPK splice variant in drug resistance was further analyzed in this study.

Facing the limitations of techniques in studying alternative splicing, our research identified the CENPK–delta8 splice variant associated with acquired resistance to Abiraterone acetate (AA) (Figure 1 and Figure S1). We assessed its functional impact on cell growth and migration using preclinical models, including patient-derived mouse xenografts (PDX), 3D organoids from patient responders and non-responders, and in vitro models, as well as clinical data from the PROMOTE study, TCGA, and TCGA SpliceSeq. Although characterizing splicing abnormalities in mCRPC human and mouse tissues is challenging and not extensively reported [53,54,55,56], our findings provide valuable insights that could guide treatment decisions and potentially improve therapeutic outcomes (Figure 6).

A repertoire of splicing isoforms has been associated with the acquired resistance [57]. For example, alternative splices *MKNK2-b* and *PKM2* promote gemcitabine resistance [58], while the p61BRAF (V600E) splice isoform reduces cancer cell sensitivity to vemurafenib [59]. The *BRCA1Δ11q* variant enhances therapeutic resistance to PARP inhibition and cisplatin [60], and the CD19 splicing isoform confers resistance to CART-19 immunotherapy [61]. To address treatment resistance, small molecules targeting the process of splicing have been reported to enter clinical trials, such as H3B-8800 (Phase I; NCT02841540), GSK525762 (Phase I; NCT03150056), ZEN003694 (Phase I/II; NCT02711956), OTX105/MK-8628 (Phase I; NCT02259114) [53]. Beyond drug resistance, splice variants also impact cell-cycle regulation, apoptosis, enhanced migration and metastatic potential, biomarkers for disease prognosis, growth-involved cellular metabolism, and evasion of immune surveillance [54,55,56,62,63,64,65].

In prostate cancer, numerous splice variants play significant roles in the development of the disease, e.g., *VEGFA*, *KLF6*, *BCL2L1*, *ERG*, *SH3GLB1*, *FGFR2*, *CCND1*, *PCSK6*, *CLK1*, *PIK3CD*, *ST6GalNAc1*, *17βHSD4*, *BCL-X*, *ERG*, and *AR* [55,56,57,66]. Among these, androgen receptor AR–V7 is the most well-known splice variant, a critical factor in prostate cancer progression. A cumulative study of 672 patients from seven clinical trials shows a significantly higher level of AR–V7 in CRPCs [67]. The AR–V7 circumvents androgen ablation and promotes cancer cell growth, resulting in resistance to androgen-deprived therapy [68]. Although splice variants underlying resistance to Abiraterone have not been extensively studied [69,70], Enzastaurin, a clinical PKCβ inhibitor, combined with an FDA-approved anti-androgen, enzalutamide, suppress both full-length AR and AR–V7, demonstrating greater efficacy in cells and xenografts [54,71]. This finding suggests the combination of splice variant inhibitor and current drug treatment may be a viable therapeutic strategy to overcome treatment resistance for precision medicine.

Despite their clinical significance, investigating splicing abnormalities, particularly in CRPC patients, poses formidable challenges [53,54,56]. These challenges include high variability among patients, cancer heterogeneity, low abundance and complexity of splicing variants, and limitations in detection technologies due to sensitivity or cost constraints [56]. Accurate quantification is also hindered by statistical and technical challenges [56]. Current methods for studying splice variants include RNA sequencing and RT–qPCR with isoform-specific primers, which we employed in this study. For RT–qPCR, well-designed primers that span splice junctions are crucial for examining mature mRNA. The CRISPR-mediated editing, such as the 3D CRISPR screen technique available in our laboratory, offers additional mechanistic insights and could be applied in future studies [27]. Detecting protein levels of splicing variants, particularly in tumor tissues, is more challenging. Although isoform-specific antibodies can be used, identifying binding-specific antibodies is a time-consuming and costly process. In our study, we were unable to obtain an antibody highly specific for CENPKΔ8. Future research should explore more approaches for protein detection, such as in situ hybridization with RNA probes, which allows for simultaneous detection of multiple isoforms with spatial resolution [72].

The CENPK is an essential component for proper kinetochore function, mitotic progression, and chromosome separation [73,74]. Recent papers report that wild-type CENPK contributes to the malignant progression, boosts proliferation and migration in gastric cancer, and predicts the prognosis of prostate cancer as well as cervical cancer [38,75]. According to the TCGA database, higher CENPK expressions are associated with shorter disease-free survival and overall survival. However, the functional role of CENPK splice variant remains poorly understood. To the best of our knowledge, our study is the first to report the involvement of the CENPK–delta8 variant in Abiraterone resistance in mCRPC.

Our study reveals that both full-length CENPK and CENPK–delta8 are present in non-mCRPC prostate cancer and mCRPC patients. Notably, mCRPC patients exhibit a higher ratio of CENPK–delta8 to full-length CENPK than primary prostate cancer patients. As shown in Figure S1, our findings indicate that (1) CENPK–delta8 is strongly correlated with AR or AR–V7, consistent with its potential role in resistance to Abiraterone; (2) high CENPK–delta8 expression is significantly correlated with high AR activity score, but not neuroendocrine score (NEPC), while CENPK all-transcripts are reversed; and (3) wild-type CENPK is highly correlated with cell-cycle progression score, a score characterizing proliferative phenotypes of the tumor, while CENPK–delta8 is moderately correlated. Additionally, CENPK–delta8 has different interaction partners, leading to CENPK variant-dependent regulation of cell migration (Figure 3). These findings align with recent literature suggesting that both full-length and splice variant play distinct yet essential roles in cell survival and proliferation [38,39].

Compared to full-length CENPK, our study reveals that FLOT1 and FLNA specifically bind to CENPK–delta8 (Figure 3 and Figure 4). The FLOT1 highly expresses in prostate tissue and its role reflects the cellular function of cytoskeleton, involving cell proliferation [11,12,13,14,15,16,17,18], cell migration and tumor progression [12,19,20], cell invasion [16], tumorigenesis [13,17], angiogenesis [76], epithelial-mesenchymal transition [17], cell-cycle modulation [14,17], and treatment resistance [11,19,21]. The knockdown of FLOT1 impairs proliferation and migration in breast cancer [12,14]. Moreover, FLOT1 promotes therapy resistance through PD–L1 immune escape via the STING signaling pathway in non-small cell lung cancer [77]. The FLOT1 has been implicated to be a prognostic biomarker in cancer [11,14,16,20,52]. Similarly, FLNA acts as a scaffold for signaling molecules and is heavily involved in cytoskeleton functions such as cell adhesion, cell movement, cell survival, the organization of extracellular matrix, metastasis, and chemoresistance [22,23,24,25]. The FLNA mutation tends to display different severity of clinical impact on females and males in that most males carrying *FLNA* mutation die prenatally or in the first years of life [78]. Further studies are needed to distinguish whether the effects observed with *FLNA* or *FLOT1* knockdown are due to a specific reversal of CENPK–delta8 impact or merely general anti-growth/migration effects.

Regulation of splicing regulatory machinery has not been well-documented [56]. Our study represents an initial effort to explore the potential of CENPKΔ8 as a biomarker. We found that CENPK–delta8 is significantly associated with several oncogenic mutants (i.e., TP53, DMD, and APC), underscoring its prognostic relevance in Abiraterone-resistant mCRPC (Figure 5). Supporting our findings, publications have shown that TP53 alteration can promote resistance to Abiraterone, rendering Abiraterone less effective in mCRPC patients with TP53 and PTEN mutations [79,80].

In this study, our ability to identify the alternative splice variant biomarkers was constrained by the limited number of patients and PDX models available in the PROMOTE study. Despite these limitations, tumors taken by mice are generally more proliferative, and our approach, using both patient tissues and corresponding PDX tumors, may help identify aggressive resistance mechanisms associated with poor outcomes [40].

According to the TCGA SpliceSeq database [81,82], genome-wide profiling of alternative splicing patterns shows that normal subjects have Exon 10 skip in *CENPK* with a percent-splice-in (PSI) value of 96.2%. However, prostate cancer patients exhibit increased exon-skipping events in the *CENPK* gene. These include Exon 10 with a PSI value of 97%, Exon 6.1 with a PSI value of 6.5%, and Exon 8 with a PSI value of 1.3%. While the CENPK Exon 8 skip event is relatively rare in patients with primary prostate cancer (1.3%), it is more prominent in those with mCRPC. Our studies provide initial evidence of the role of CENPK exon-skipping variant in mCRPC tumor growth and Abiraterone resistance.

## Data Availability

The TCGA and TCGA SpliceSeq data are available and based upon public data extracted from TCGA research network. Available online: https://www.cancer.gov/ccg/research/genome-sequencing/tcga, accessed on 1 March 2022. Patient RNA-Seq data has been deposited into the database of Genotypes and Phenotypes (dbGaP) with accession number phs001141.v1. p1.

## Supplementary Materials

The following supporting information can be downloaded at: https://www.mdpi.com/article/10.3390/cells13191622/s1, Table S1: Primer list; Table S2: IP–MS binding protein; Figure S1: The related pathways of the splice variants and their presence in 3D organoids and cell lines; Figure S2: IP–MS validation by Western blotting; Figure S3: Cytotoxicity assays of splice variant-OE in 22Rv1; Figure S4: Genes associated with CENPK splice variants by mutation or truncation; Figure S5: qRT–PCR validation of CENPK knockdown and overexpression efficiency; Figure S6: Representative image of wild-type CENPK and CENPK–delta8 overexpression; Figure S7: qRT–PCR validation of knockdown efficiency for the clinically relevant genes.
