# Supplementary material for: The Role of CENPK Splice Variant in Abiraterone Response in Metastatic Castration-Resistant Prostate Cancer
Source: Cells. 2024 Sep 28;13(19):1622. doi: 10.3390/cells13191622 (PMC11475995; doi:10.3390/cells13191622)
Supplement: Supplementary file 1 [file cells-13-01622-s001.zip › cells-3169742-supplementary figures.pdf]

Supplementary Materials

Research Article

The role of CENPK splice variant in Abiraterone response in metastatic castration-resistant prostate cancer

Minhong Huang [1], Sisi Qin [2], Huanyao Gao [1], Wootae Kim [3], Fang Xie [4], Ping Yin [1], August John [1], Richard M. Weinshilboum [1], Liewei Wang [1,*]

[1] Department of Molecular Pharmacology and Experimental Therapeutics, Mayo Clinic, Rochester, MN, USA
[2] Department of Pathology, University of Chicago, Chicago, IL, USA
[3] Department of Integrated Biomedical Science, Soonchunhyang Institute of Medi-Bio Science (SIMS), Soonchunhyang University, Cheonan, Chungcheongnam-do, Republic of Korea
[4] Division of Medical Oncology and Cancer Center, Beth Israel Deaconess Medical Center, Boston, MA
* Correspondence: Liewei Wang, M.D., Ph.D., Bernard and Edith Waterman Director and Department Chair, 200 First Street Southwest, Rochester, MN, 55905. Telephone: (507) 284-5264; Fax: (507) 284-4455; email: Wang. Liewei@mayo.edu

A

#Chrom	Splice_start	Splice_end	Gene	Event	Related pathways
chr5	64824405	64824943	CENPK	Skip exon-8	Cell cycle, mitotic
chrX	153607922	153608049	EMD	Use cryptic 5' splice site, 4 nt deletion	Cell cycle, mitotic
chr16	2135323	2136240	TSC2	Use cryptic 3' splice site, 47 nt deletion	PI3K-Akt pathway, apoptosis
chr19	14562835	14574478	PKN1	Skip exon-8 and exon-9	PI3K-Akt pathway, apoptosis
chr1	109716179	109716309	KIAA1324	Alternative splicing (use isoform uc009wey. 3)	Tumor progression and metastasis

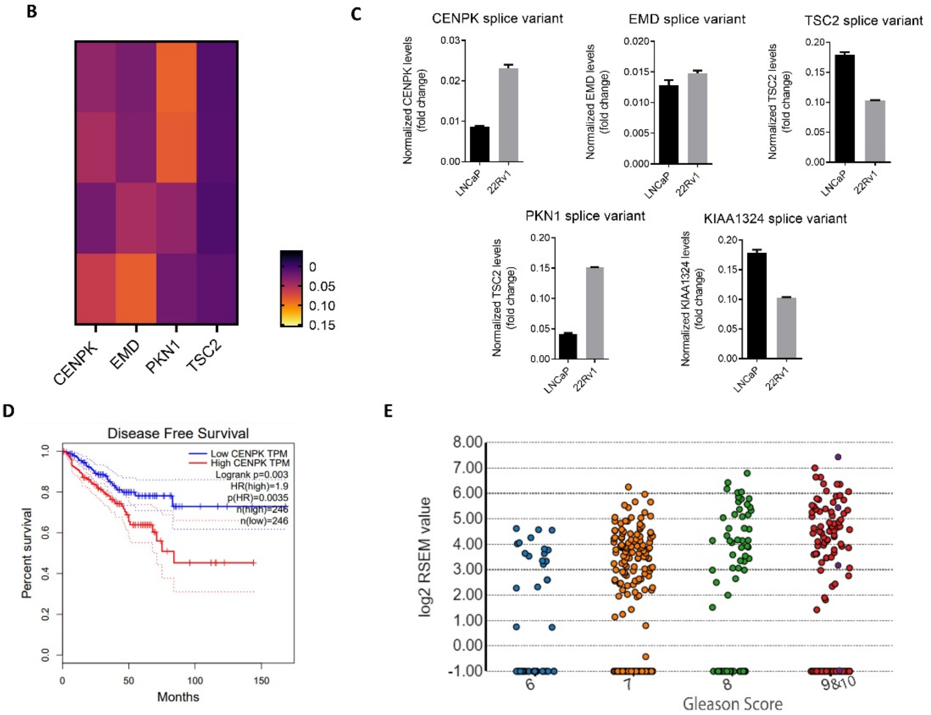

Supplementary Figure 1. The related pathways of the splice variants and their presence in 3D organoids and cell lines. (A) The related pathways of the significant splice variants in mCRPC patients, detected from PROMOTE clinical study. (B) qRT-PCR heat map of representative splice variants in 3D organoids including CENPK, EMD, TSC2, and PKN1 . The first row is CENPK gene expression; the second one is EMD; the third is PKN1 while the fourth is TSC2. The colors represent the gene expression levels. Light yellow is highest expression while dark purple is the lowest expression. This was to validate their presence in responder and non-responder 3D organoids. (C) qRT-PCR validation of CENPK, EMD, TSC2, PKN1, and KIA1324 splice variants in LNCaP and22Rv1 cell lines. (D) Disease-free survival analysis (in months) in CENPK high expression vs. low expression prostate cancer patients from TCGA database. (E) The clinical correlation between Gleason stage and CENPK-delta8 expression (in RSEM) based on TCGA SpliceSeq database (the web tool TSVdb was used for splicing variants analysis). Gleason scores range from 2-10. A Gleason score of 6 is low grade, 7 is intermediate grade, and a score of 8 to 10 is high grade cancer.

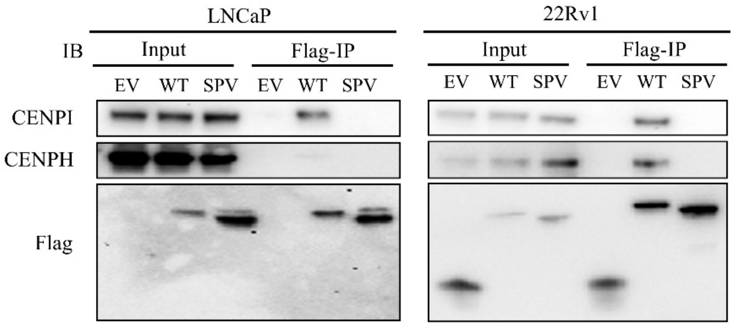

Supplementary Figure 2. IP-MS validation by Western Blotting. Western blots of overexpressed CENPK-delta8 and overexpressed wild-type CENPK in LNCaP (the left) and 22Rv1 (the right) cells. Their corresponding cells with overexpressed empty vector were as control and input as comparison. Flag was the tag used to pull down the CENPK complex. CENPI and CENPH are the components of the CENPK complex.

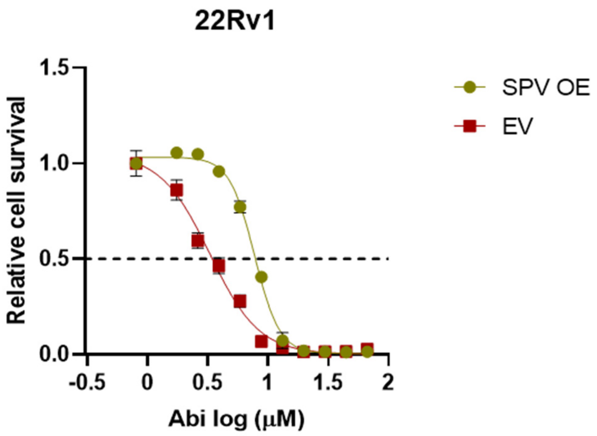

Supplementary Figure 3. Cytotoxicity assays of splice variant-OE in 22Rv1. SPV, CENPK splice variant or CENPK-delta8; KD, knockdown.

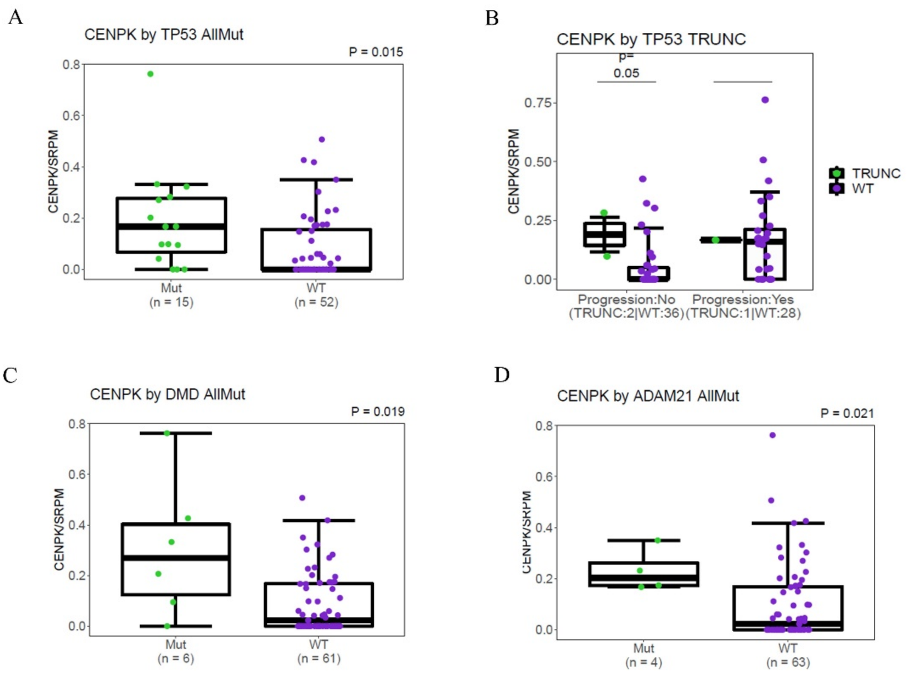

Supplementary Figure 4. Genes associated with CENPK splice variants by mutation or truncation. Data analysis was based on the patient cohort from PROMOTE study (Mayo Clinic).

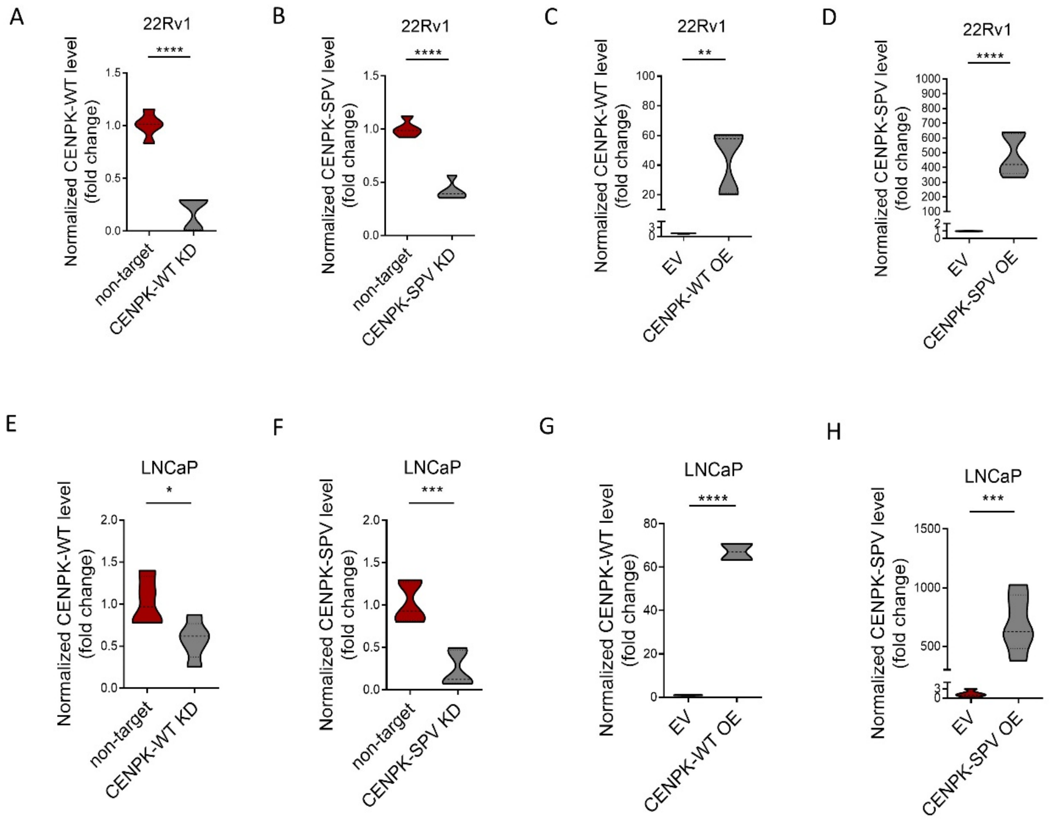

Supplementary Figure 5. qRT-PCR validation of CENPK knockdown and overexpression efficiency. Wild-type CENPK and CENPK-delta8 knockdown and overexpression in (A-D) 22Rv1, in (E-H) DU145, in (I-L) LNCaP, and in (M-P) PC3. EV, empty vector; WT, wild-type CENPK; SPV, CENPK splice variant; KD, knockdown; OE, overexpression. Statistical significance indicated unpaired t- test: *P < 0.05, **P < 0.01, ***P <0.001, ****P <0.0001.

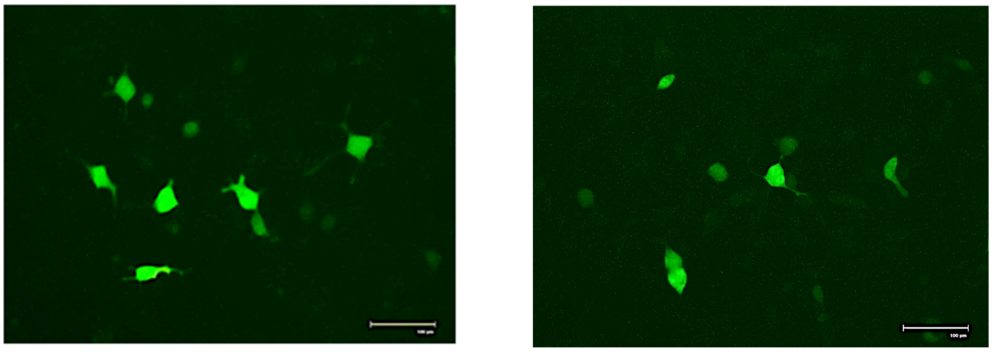

Supplementary Figure 6. Representative image of wild-type CENPK and CENPK-delta8 overexpression, showing GFP signal in WT OE and SPV OE prostate cells. Scale bar, 100 µm. WT, wild type. SPV, splice variant.

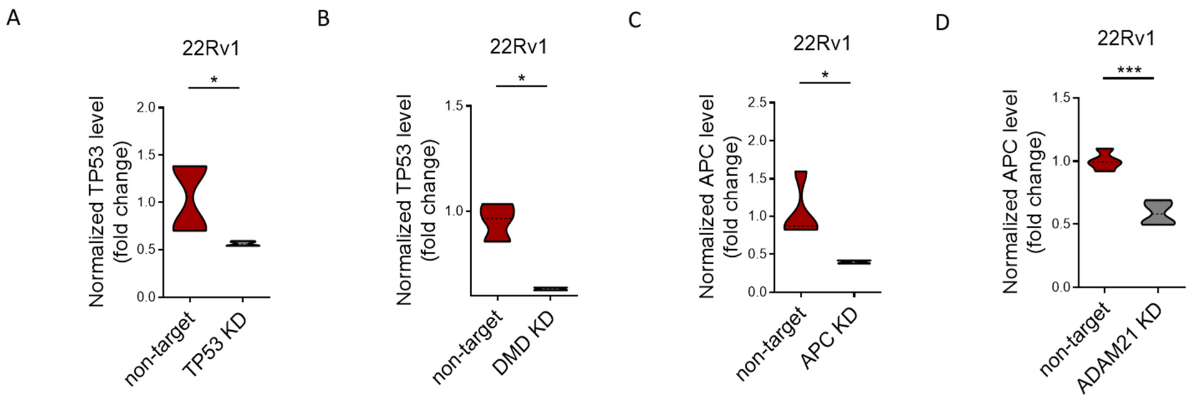

Supplementary Figure 7. qRT-PCR validation of knockdown efficiency for the clinically relevant genes. TP53, DMD, APC, and ADAM21 knockdown in KD, knockdown. Statistical significance indicated unpaired t- test: *P < 0.05, **P < 0.01, ***P <0.001, ****P <0.0001.
