# Supplementary material for: The Role of CENPK Splice Variant in Abiraterone Response in Metastatic Castration-Resistant Prostate Cancer
Source: Cells. 2024 Sep 28;13(19):1622. doi: 10.3390/cells13191622 (PMC11475995; doi:10.3390/cells13191622)
Supplement: Supplementary file 1 [file cells-13-01622-s001.zip › Supplementary Table 1_Primer list.pdf]

Supplementary Table 1. Primers and siRNAs sequences

Name	Information	Source
**Primers for target genes**		
TP53_F	CCCCTCTGAGTCAGGAAACA	IDT
TP53_R	GGGACAGCATCAAATCATCC	
DMD_F	TGGGCAAACTGTATTCACTCAAAC	
DMD_R	TTCCCTTGTGGTCACCGTAGT	
RYR2_F	TGATGCTGCTGACTGCTCTT	
RYR2_R	ATTGGGTACTTGCCCCTTCT	
KMT2C_F	GATTTTATGCAACCTCTAGGACC	
KMT2C_R	GCTTCACAGAAGAAAAATTTGG	
GAPDH_F	ACATCGCTCAGACACCATG	
GAPDH_R	TGTAGTTGAGGTCAATGAAGGG	
FLNA_F	CAACAAGTTCACTGTGGAGACCA	
FLNA_R	TGTAGGTGCCAGCCTCATAAGG	
CENPK-SPV_F	GACTTAGAAAGAATCTTTAATGAACTG	
CENPK-SPV_R	CATGCAGTGTTATCAGGTTTA	
CENPK_F	GGGAACAACGGTGGTTGGATG	
CENPK_R	CTTCTAGAAACTCGCCCAAGGTACTC	
**Primers for target genes**		
ADAM21	QuantiTect Primer Assay®, Cat. No. QT00209699	QIAGEN
**siRNAs for knocking down target genes**		
SMARTpool FLNA siRNA	GCAGGAGGCUGGCGAGUAU	Horizon Discovery
	GCACCCAGACCGUCAAUUA	
	GCACAUGUUCCGUGUCCUA	
	GAAUGGCGUUUACCUGAUU	
SMARTpool FLOT1 siRNA	GCAGAGAAGUCCCAACUAA	Horizon Discovery
	GUGGUUAGCUACACUCUGA	
	GAUCAGUGGUCCCUUGACU	
	GAAGACGGAGGCUGAGAUU	
SMARTpool CENPK siRNA	GAAGAUCCAACCCGAAUAA	Horizon Discovery
	CUAAGGAGUCAAAGAAUGA	
	CGAUUCAAAUGCUCAGCUA	
	GCGAGUUUCUAGAAGACCA	
Customized CENPK-SPV siRNA	Sense: CUUAGAAAGAAUCUUUAAUGUUUU	Horizon Discovery
	Antisense: AACAUUAAAGAUUCUUUCUAAGUU	
Non-Targeting Control	UGGUUUACAUGUCGACUAA	Horizon Discovery
